# TGF-β1 induces VEGF expression in human granulosa-lutein cells: a potential mechanism for the pathogenesis of ovarian hyperstimulation syndrome

**DOI:** 10.1038/s12276-020-0396-y

**Published:** 2020-03-10

**Authors:** Lanlan Fang, Yiran Li, Sijia Wang, Yuxi Li, Hsun-Ming Chang, Yuyin Yi, Yang Yan, Avinash Thakur, Peter C. K. Leung, Jung-Chien Cheng, Ying-Pu Sun

**Affiliations:** 1 Center for Reproductive Medicine, Henan Key Laboratory of Reproduction and Genetics, The First Affiliated Hospital of Zhengzhou University, 450052 Zhengzhou, Henan China; 2 Department of Obstetrics and Gynaecology, BC Children’s Hospital Research Institute, University of British Columbia, Vancouver, BC V5Z 4H4 Canada; 3 Terry Fox Laboratory, BC Cancer Agency, Vancouver, BC V5Z 1L3 Canada

**Keywords:** Endocrine reproductive disorders, Experimental models of disease, Infertility

## Abstract

Ovarian hyperstimulation syndrome (OHSS) is one of the most serious and iatrogenic complications that can occur during in vitro fertilization treatment. Although the pathogenesis of OHSS is not fully understood, vascular endothelial growth factor (VEGF) has been recognized as an important mediator of the development of OHSS. Transforming growth factor-beta-1 (TGF-β1) is known to regulate various ovarian functions. However, whether VEGF can be regulated by TGF-β1 in human granulosa cells has not been determined. In addition, the role of TGF-β1 in the pathogenesis of OHSS remains unknown. In the present study, we demonstrate that TGF-β1 stimulates VEGF expression in and secretion from both immortalized human granulosa-lutein (hGL) cells and primary hGL cells. Our results demonstrate that the SMAD2/3, ERK1/2, and p38 MAPK signaling pathways are involved in TGF-β1-induced VEGF expression and secretion. Using a mouse OHSS model, we show that the expression levels of TGF-β1 and VEGF are increased in the ovaries of OHSS mice. Blocking TGF-β1 signaling inhibits the development of OHSS by attenuating VEGF expression. Moreover, clinical results reveal that the protein levels of TGF-β1 and VEGF are increased in the follicular fluid of patients with OHSS, and that the levels of these two proteins in the follicular fluid are positively correlated. The results of this study help to elucidate the mechanisms by which VEGF expression is regulated in hGL cells, which could lead to the development of alternative therapeutic approaches for treating OHSS.

## Fertility treatment: Understanding ovarian hyperstimulation syndrome

Insights into abnormal protein activity involved in ovarian hyperstimulation syndrome (OHSS) could inform future therapies. OHSS is a serious complication of fertility treatment where the body responds excessively to medication taken to help eggs grow, causing blood clots, pain, and even death. Scientists believe that high levels of the vascular endothelial growth factor (VEGF) protein trigger OHSS. Another protein involved in cell proliferation,transforming growth factor beta-1 (TGF-β1), may influence VEGF activity. Ying-Pu Sun and Jung-Chien Cheng at the First Affiliated Hospital of Zhengzhou University, Henan, China, and co-workers conducted experiments on human cell lines and OHSS mouse models. They found that TGF-β1 stimulates VEGF expression in human cells, and identified the associated signaling pathways. Both TGF-β1 and VEGF were increased in the ovaries of mice with OHSS, while blocking TGF-β1 halted OHSS progression.

## Introduction

Ovarian hyperstimulation syndrome (OHSS) is one of the most serious and iatrogenic complications resulting from ovarian stimulation with exogenous gonadotropins and ovulation induction by human chorionic gonadotropin (hCG) during in vitro fertilization (IVF) treatment^1^. The incidence of mild, moderate, and severe OHSS within all IVF cycles is 20%–33%, 3%–8%, and 0.1%–3%, respectively. Although the incidence of severe OHSS is low, it can be life-threatening^2^. The symptoms of OHSS include massively enlarged ovaries, ascites, hydrothorax, renal failure, venous embolism, and even death. It has been well established that the critical characteristic of OHSS is increased capillary permeability, which leads to a fluid shift from the intravascular space to third space areas^3^.

The transforming growth factor-beta (TGF-β) superfamily is composed of TGF-βs, activins/inhibins, anti-Mullerian hormone (AMH), bone morphogenetic proteins (BMPs), growth and differentiation factors (GDFs), and other proteins that have been shown to regulate various physiological and pathological events in the ovary^4^. Immunohistochemical analyses of human ovarian tissue show that TGF-β1 protein expression can be detected in both granulosa and theca cells, whereas TGF-β2 is specifically localized in the theca cells of ovarian follicles^5,6^. In addition, both TGF-β receptor type I (TβRI) and type II (TβRII) are expressed in human granulosa cells^7^. Importantly, TGF-β1 protein can be detected in the human follicular fluid^8,9^, which indicates that granulosa cell-secreted TGF-β1 may play important autocrine/paracrine roles in the regulation of ovarian functions.

Vascular endothelial growth factor (VEGF) was originally described as an endothelial cell-specific mitogen. VEGF can increase vascular permeability and stimulate angiogenesis^10^. VEGF acts as a key vasoactive factor in inducing OHSS, as incubation of ascitic fluid from hyperstimulated women with VEGF antiserum significantly decreases vascular permeability in a guinea pig model^11^. After hCG injection, the levels of VEGF in the follicular fluid and serum become greatly increased in OHSS patients. Interestingly, the levels of VEGF in follicular fluid are considerably higher than they are in serum^12^. VEGF and its receptors are expressed in the granulosa cells of preovulatory follicle and in the granulosa-lutein cells of the corpus luteum^13–15^. Our group and other groups have shown that treatment of human granulosa-lutein (hGL) cells with hCG upregulates the expression of VEGF^16–18^. Importantly, studies in both different animal models and humans have shown that targeting VEGF or its receptor can prevent the development of OHSS^19,20^. Altogether, these studies indicate that locally produced VEGF in the ovary is an important factor that mediates the pathogenesis of OHSS.

It has been reported that few cytokines and growth factors, including TGF-β1, can increase in VEGF protein levels and induce its secretion in different types of cells^21^. Our previous studies have demonstrated that TGF-β1 can regulate steroidogenesis, cell proliferation, and differentiation in hGL cells^22–25^. A previous study showed that TGF-β1 increases the secretion of VEGF and stimulates angiogenic activity in rat granulosa cells^26^. However, whether the same effect is true for human granulosa cells remains unknown. In addition, AMH is a member of the TGF-β superfamily, and its levels in serum and follicular fluid are significantly higher in OHSS patients than in patients without OHSS^27^. Whether the levels of TGF-β1 are different between non-OHSS and OHSS patients has not been determined. In the present study, we aimed to examine the effect and the underlying molecular mechanisms of TGF-β1 on VEGF expression in hGL cells. We also explored the role of TGF-β1 in OHSS pathogenesis in mice.

## Materials and methods

### Cell cultures and reagents

A nontumorigenic SV40 large T-antigen immortalized human granulosa cell line (SVOG) that was established previously by our group was used in the present study^28^. Primary cultures of human granulosa-lutein (hGL) cells were purified by density centrifugation from follicular aspirates collected from women undergoing oocyte retrieval, as previously described^29^. SVOG and hGL cells were grown in DMEM/F12 medium (Gibco, Shanghai, China) supplemented with 10% charcoal/dextran-treated fetal bovine serum (FBS) (HyClone, Shanghai, China), 100 U/mL penicillin and 100 μg/mL streptomycin (Boster, Wuhan, China). The cultures were maintained at 37 °C in a humidified atmosphere of 5% CO_2_. Recombinant human TGF-β1 was obtained from R&D Systems (Shanghai, China). Pregnant mare serum gonadotropin (PMSG) was obtained from Solarbio (Beijing, China). hCG was obtained from Livzon (Zhuhai, China). SB431542, SB203580 and U0126 were obtained from Sigma (Shanghai, China). Monoclonal anti-phospho-ERK1/2 (Thr202/Tyr204) and polyclonal anti-SMAD4, anti-ERK1/2, anti-phospho-p38 (Thr180/Tyr182), and anti-p38 antibodies were obtained from Cell Signaling (Shanghai, China).

### Patients and follicular fluid collection

The studies that used clinical samples were approved and were carried out in accordance with the guidelines from the Zhengzhou University Research Ethics Board. High risk OHSS patients were selected based on age, a low body mass index (BMI), polycystic ovarian morphology and a previous history of high responsiveness to gonadotrophins^30^. Following gonadotropin treatment and oocyte retrieval, 30 patients diagnosed with severe OHSS were recruited. These patients did not undergo fresh embryo transfer. Another 30 infertile patients without OHSS were randomly selected as a control group that was matched by age, body mass index (BMI) and serum levels of basal FSH, LH, estradiol (E2), progesterone (P4), and prolactin (PRL) (Supplemental Table 1). For all patients, the inclusion criteria were as follows: age between 20 and 35 years, BMI between 19 and 24.9, regular menstrual cyclicity, tubal factor infertility or male factor fertility, and no complications, such as diabetes or abnormal thyroid function. The exclusion criteria were as follows: polycystic ovarian syndrome, endometriosis, diminished ovarian reserve, chromosome abnormality or hydrosalpinx. All patients were treated with a standard long protocol. At the mid-luteal phase, 0.1 mg of the gonadotropin-releasing hormone (GnRH) agonist triptorelin (Ipsen Pharma Biotech, Boulogne-Billancourt, France) was subcutaneously administered daily. Approximately 14 days after the injection of GnRH agonist, 150–300 IU recombinant FSH (Gonal-F; Merck, Germany) was administered daily. When at least three follicles had reached 18 mm, hCG (10,000 IU, Livzon, Zhuhai, China) was injected. Oocyte retrieval was scheduled at approximately 34–36 h after hCG injection by transvaginal ultrasound-guided follicular aspiration. The follicular fluid was collected when the oocytes were retrieved. Only the first follicular fluid aspirate without blood or flushing solution was used for analysis. After 10 min of centrifugation at 1200 rpm, the supernatant was stored at −80 °C until further analysis.

### Reverse transcription quantitative real-time PCR (RT-qPCR)

Total RNA was extracted using TRIzol reagent (Invitrogen, Shanghai, China) according to the manufacturer’s instructions. RNA (2 μg) was reverse-transcribed to generate first-strand complementary DNA (cDNA) with a GoldScript one-step RT-PCR kit (Applied Biosystems, Shanghai, China). Each 20 μL qPCR reaction contained 1X SYBR Green PCR Master Mix (Applied Biosystems), 20 ng of cDNA and 250 nM of each specific primer. The primers used for RT-qPCR were the following: VEGF, 5′-CCC ACT GAG GAG TCC AAC AT-3′ (sense) and 5′-TGC ATT CAC ATT TGT TGT GC-3′ (antisense); TGF-β receptor I, 5′-GTT AAG GCC AAA TAT CCC AAA CA-3′ (sense) and 5′-ATA ATT TTA GCC ATT ACT CTC AAG G-3′ (antisense); TGF-β receptor II, 5′-TGT GGA TGA CCT GGC TAA CA-3′ (sense) and 5′-TCG GTC TGC TTG AAG GAC TC-3′ (antisense); SMAD2, 5′-GCC TTT ACA GCT TCT CTG AAC AA-3′ (sense) and 5′-ATG TGG CAA TCC TTT TCG AT-3′ (antisense); SMAD3, 5′-CCC CAG CAC ATA ATA ACT TGG-3′ (sense) and 5′- AGG AGA TGG AGC ACC AGA AG-3′ (antisense); SMAD4, 5′-TGG CCC AGG ATC AGT AGG T-3′ (sense) and 5′-CAT CAA CAC CAA TTC CAG CA-3′ (antisense); GAPDH, 5′-GAG TCA ACG GAT TTG GTC GT-3′ (sense) and 5′-GAC AAG CTT CCC GTT CTC AG-3′ (antisense); mouse VEGF, 5′-GAC CCT GGC TTT ACT GCT GT-3′ (sense) and 5′-AGA TGT CCA CCA GGG TCT CA-3′ (antisense); mouse TGF-β1, 5′-AGT GTG GAG CAA CAT GTG GA-3′ (sense) and 5′-TGC CGT ACA ACT CCA GTG AC-3′ (antisense) and mouse GAPDH, 5′-TTG TGG AAG GGC TCA TGA-3′ (sense) and 5′-GAT GCA GGG ATG ATG TTC-3′ (antisense). RT-qPCR was performed using an Applied Biosystems QuantStudio 12 K Flex Real-Time PCR system equipped with a 96-well optical reaction plate. The specificity of each assay was validated by melting curve analysis and agarose gel electrophoresis of the PCR products. All of the RT-qPCR experiments were run in triplicate, and a mean value was used to determine the messenger RNA (mRNA) levels. Water and mRNA without RT were used as negative controls. Relative quantification of the mRNA levels was performed using the comparative Ct method; GAPDH was the reference gene, and the 2^–∆∆Ct^ formula was used.

### Western blot

Cells were lysed in cell lysis buffer (Cell Signaling Technology). Equal amounts of protein were separated by sodium dodecyl sulfate polyacrylamide gel electrophoresis and then were transferred onto polyvinylidene fluoride membranes. After 1 h of blocking with 5% nonfat dry milk in Tris-buffered saline (TBS), the membranes were incubated overnight at 4 °C with primary antibodies diluted in 5% nonfat milk/TBS. Following primary antibody incubation, the membranes were incubated with the appropriate HRP-conjugated secondary antibodies. Immunoreactive bands were detected using an enhanced chemiluminescent substrate (Bio-Rad Laboratories (Shanghai, China) and were imaged with a ChemiDoc MP Imager (Bio-Rad Laboratories).

### Small-interfering RNA (siRNA) transfection

To knock down endogenous TGF-β receptor I, TGF-β receptor II, SMAD2, SMAD3 and SMAD4, cells were transfected with 50 nM ON-TARGETplus SMARTpool siRNAs targeting each specific gene (Dharmacon, Lafayette, CO) using Lipofectamine RNAiMAX (Invitrogen). A siCONTROL NON-TARGETING pool siRNA (Dharmacon) was used as a transfection control. The knockdown efficiency was examined by using RT-qPCR.

### Measurement of VEGF and TGF-β1 levels

The levels of human VEGF in culture media and follicular fluid samples were measured according to the manufacturer’s instructions using a VEGF enzyme-linked immunosorbent assay (ELISA) kit (R&D Systems). The levels of human TGF-β1 in follicular fluid samples were measured according to the manufacturer’s instructions using a TGF-β1 enzyme-linked immunosorbent assay (ELISA) kit (R&D Systems).

### Chromatin immunoprecipitation (ChIP)

The ChIP assay was performed with the use of the ChIP-IT Express Enzymatic kit (Active Motif, Shanghai, Carlsbad, CA) according to the manufacturer’s protocol. The purified DNA was subjected to PCR amplification (one cycle of 94 °C for 3 min and 40 cycles of 94 °C for 20 s, 64 °C for 30 s and 72 °C for 30 s) of the SMAD4 binding site within the VEGF promoter using specific forward (5′-AGC TGA GAC GAA ACC CCC AT-3′) and reverse (5′- GGA AGA GGA CCT GTT GGA GC-3′) primers. The selected primers were confirmed with an in silico PCR program (http://genome.cse.ucsc.edu/cgi-bin/hgPcr) to ensure the generation of a single amplicon from the human genomic DNA. The PCR products (320 bp) were resolved by electrophoresis on a 1% agarose gel and then were visualized by ethidium bromide staining.

### Mouse OHSS model

Female ICR mice were obtained from Charles River Laboratories (Beijing, China). Animal handling was performed in accordance with the Guide for the Care and Use of Laboratory Animals published by the US National Institutes of Health. The mice were housed in an environmentally controlled room and had free access to food and water. Animal studies were approved by the Zhengzhou University Animal Research Ethics Board. The mouse OHSS model was established according to a previous study with minor modifications^31^. PMSG (20 IU/day) was administered i.p. for 4 consecutive days to 5-week-old ICR female mice, and this was followed by hCG administration (7 IU, i.p.) on the fourth day. Control mice were administered a single dose of PMSG (5 IU), which was followed by hCG (7 IU) administration 48 h later. Mice were treated with a vehicle control (DMSO) or SB431542 (10 mg/kg, i.p.) on days 4–6. All mice were euthanized on day 7. Each group contained ten mice. Changes in body weight and ovarian weight were recorded.

### Statistical analysis

The results are presented as the mean ± SEM or mean ± SD of at least three independent experiments. Prism 8 software was used for all statistical analyses. For experiments involving only two groups, the data were analyzed by *t*-test. Multiple group comparisons were analyzed by one-way ANOVA followed by Tukey’s multiple comparison test. A significant difference was defined as *p* < 0.05.

## Results

### TGF-β1 stimulates VEGF expression in and secretion from human granulosa-lutein cells

**Fig. 1:**
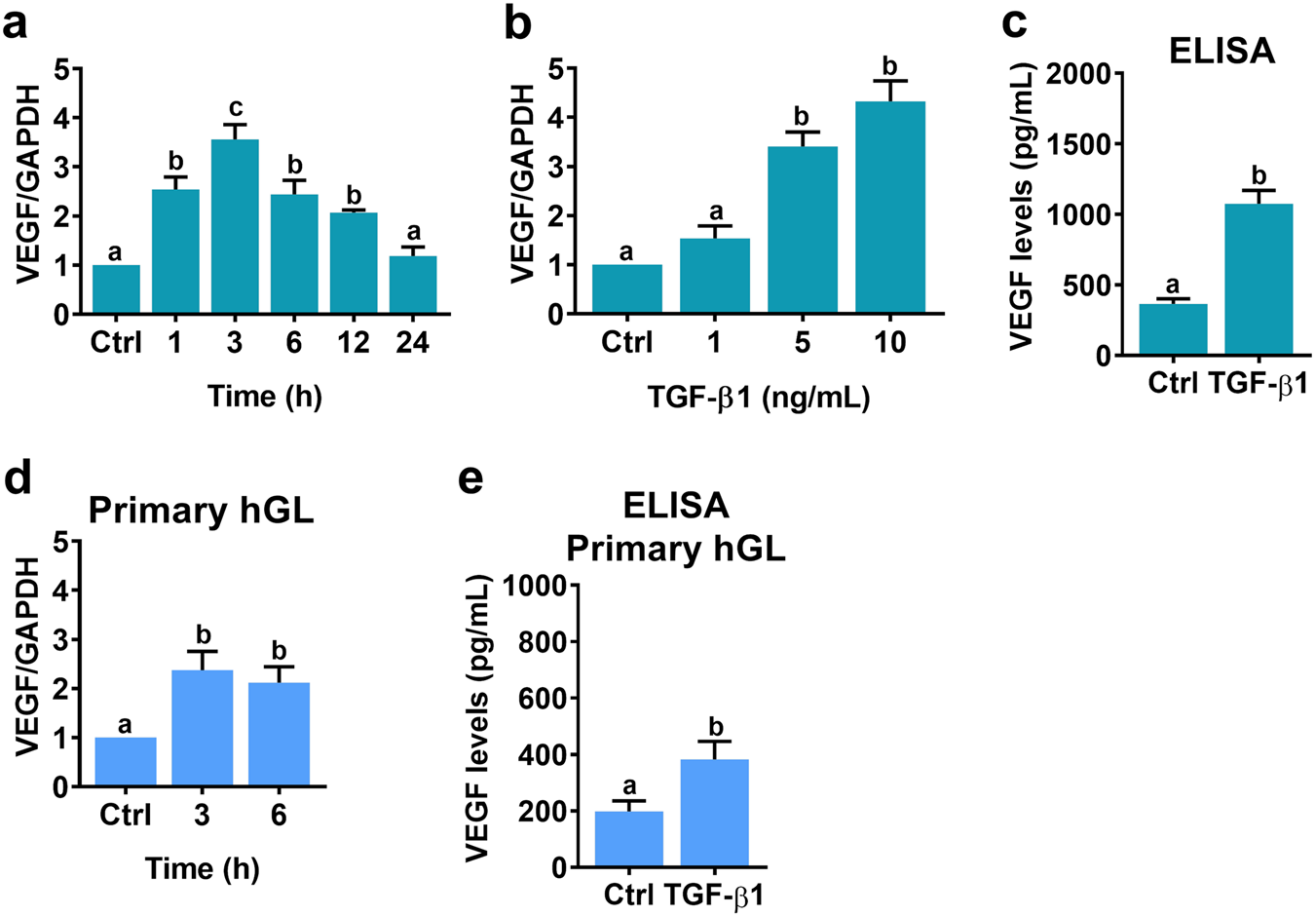
TGF-β1 stimulates VEGF expression in SVOG and primary hGL cells. **a** SVOG cells were treated with 5 ng/mL TGF-β1 for different periods of time, and the mRNA levels of VEGF were examined by RT-qPCR. The level of VEGF mRNA at each time point was normalized to the GAPDH mRNA level at the same time point. **b** SVOG cells were treated with 1, 5, or 10 ng/mL TGF-β1 for 6 h, and the mRNA levels of VEGF were examined by RT-qPCR. **c** SVOG cells were treated with 5 ng/mL TGF-β1 for 24 h, and the protein levels of VEGF in culture media were examined by ELISA. **d** Primary hGL cells were treated with 5 ng/mL TGF-β1 for 3 and 6 h, and then the mRNA levels of VEGF were examined by RT-qPCR. **e** Primary hGL cells were treated with 5 ng/mL TGF-β1 for 24 h, and the protein levels of VEGF in culture media were examined by ELISA. The results are expressed as the mean ± SEM of at least three independent experiments. The values without a common letter are significantly different (*p* < 0.05).

We established an SV40 large T-immortalized primary human granulosa-lutein cell line, which was named SVOG^28^. Using this cell model, we previously successfully demonstrated several important biological functions of TGF-β1^23–25^. In the present study, we continued to use this cell model to examine the effect of TGF-β1 on VEGF expression. As shown in Fig. 1a, time-course experiments showed that treatment of SVOG cells with 5 ng/mL TGF-β1 significantly increased VEGF mRNA levels after 1 h. The most significant effect was observed after 3 h of TGF-β1 treatment; the levels then decreased but remained detectable after 12 h of treatment. In addition, we also examined the effect of different concentrations of TGF-β1 on VEGF expression. As shown in Fig. 1b, treatment with 1 ng/mL TGF-β1 did not significantly increase VEGF mRNA levels. Treatment with 5 ng/mL or 10 ng/mL TGF-β1 induced a comparable increase in VEGF mRNA levels. Therefore, 5 ng/mL TGF-β1 was used in all subsequent experiments. To examine the levels of secreted VEGF, culture media from SVOG cells treated with TGF-β1 were collected, and the protein levels of VEGF were measured by ELISA. As shown in Fig. 1c, TGF-β1 significantly stimulated the secretion of VEGF protein. Similar results were obtained by performing the same experiments in primary cultures of human granulosa-lutein (hGL) cells (Fig. 1d, e).

### TGF-β receptors mediate TGF-β1-induced VEGF expression in and secretion from human granulosa-lutein cells

**Fig. 2:**
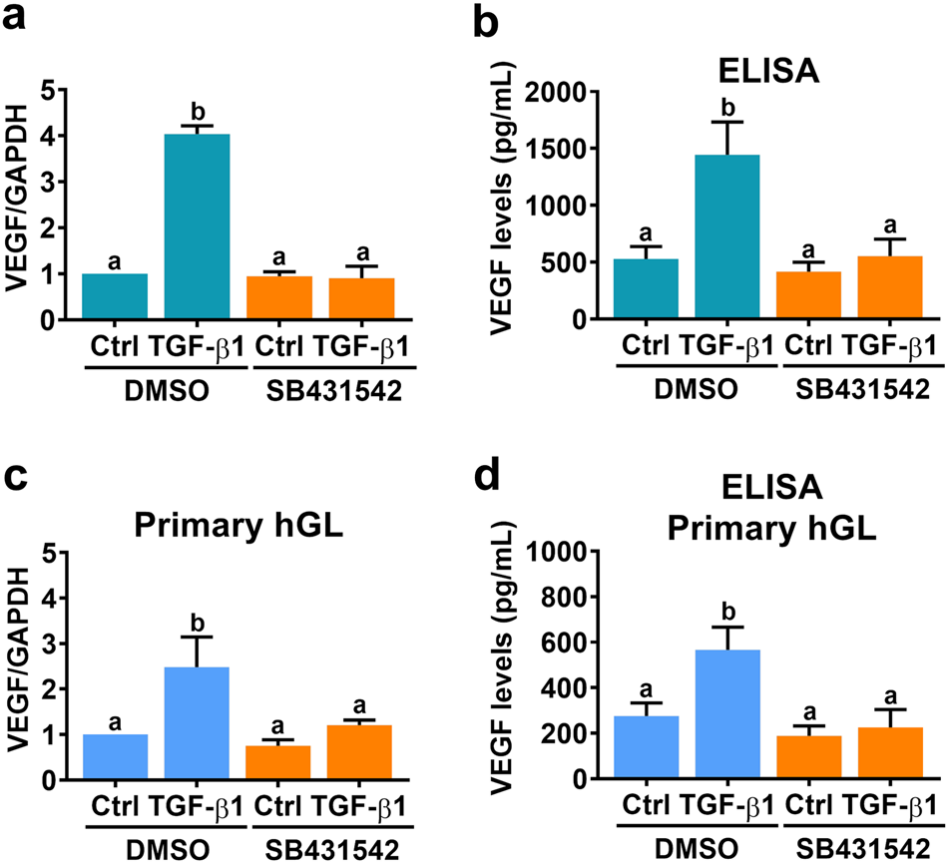
Pharmacological inhibition of TGF-β receptor I blocks TGF-β1-induced VEGF expression and secretion. **a** SVOG cells were pretreated with a vehicle control (DMSO) or 10 µM SB431542 for 1 h, and then they were treated with 5 ng/mL TGF-β1 for 6 h. VEGF mRNA levels were examined by RT-qPCR. **b** SVOG cells were pretreated with a vehicle control (DMSO) or 10 µM SB431542 for 1 h, and then they were treated with 5 ng/mL TGF-β1 for 24 h. VEGF protein levels in culture media were examined by ELISA. **c** Primary hGL cells were pretreated with a vehicle control (DMSO) or 10 µM SB431542 for 1 h, and then they were treated with 5 ng/mL TGF-β1 for 6 h. VEGF mRNA levels were examined by RT-qPCR. **d** Primary hGL cells were pretreated with a vehicle control (DMSO) or 10 µM SB431542 for 1 h, and then they were treated with 5 ng/mL TGF-β1 for 24 h. VEGF protein levels in culture media were examined by ELISA. The results are expressed as the mean ± SEM of at least three independent experiments. The values without a common letter are significantly different (*p* < 0.05).

**Fig. 3:**
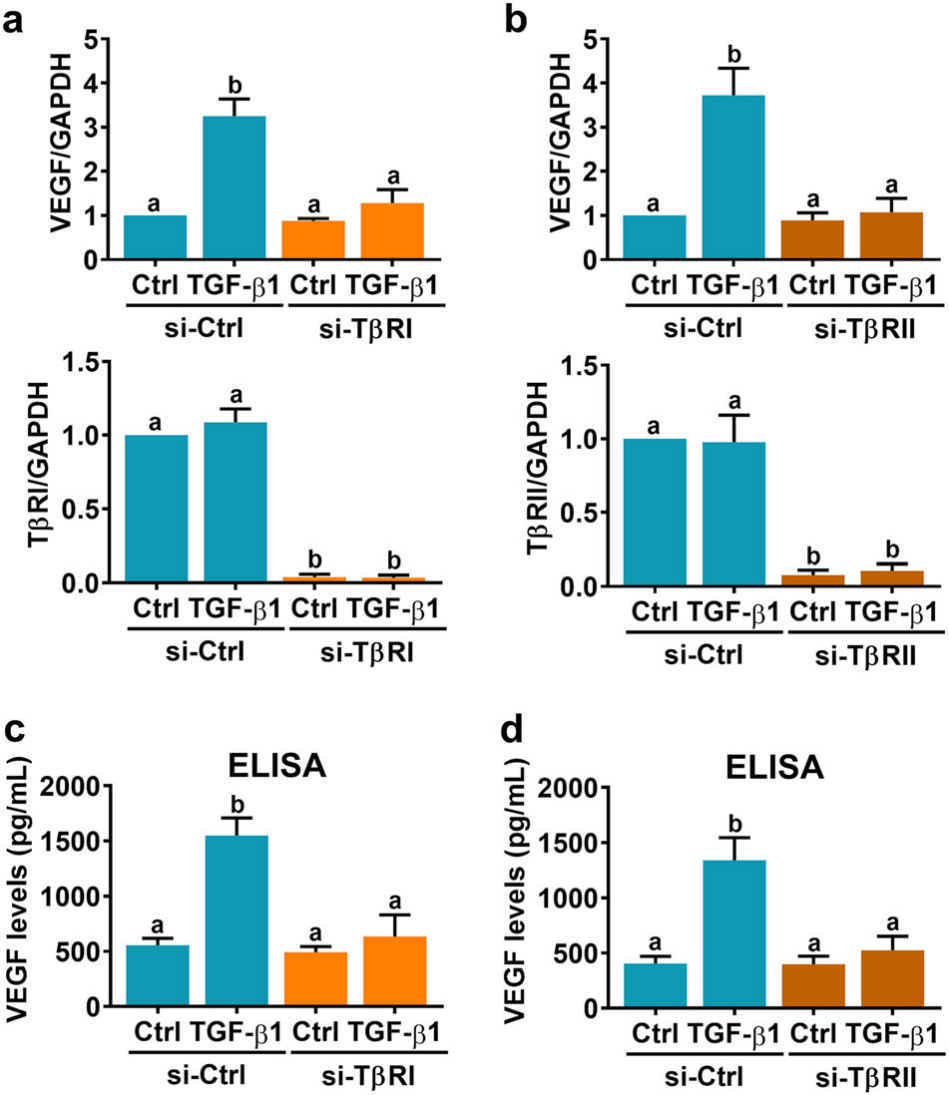
Knockdown of TGF-β receptor I and II blocks TGF-β1-induced VEGF expression and secretion. **a**, **b** SVOG cells were transfected with 50 nM control siRNA (si-Ctrl), TGF-β receptor I siRNA (si-TβRI) (**a**) or TGF-β receptor II siRNA (si-TβRII) (**b**), and after 48 h they were treated with 5 ng/mL TGF-β1 for 6 h. VEGF, TβRI, and TβRII mRNA levels were examined by RT-qPCR. **c**, **d** SVOG cells were transfected with 50 nM control siRNA (si-Ctrl), TGF-β receptor I siRNA (si-TβRI) (**c**) or TGF-β receptor II siRNA (si-TβRII) (**d**), and after 48 h they were treated with 5 ng/mL TGF-β1 for 24 h. Protein levels of VEGF in culture media were examined by ELISA. The results are expressed as the mean ± SEM of at least three independent experiments. The values without a common letter are significantly different (*p* < 0.05).

TGF-β1 activates downstream signaling pathways and regulates biological functions by binding to the transmembrane receptors TβRI and TβRII^32^. To examine the requirement of TGF-β receptors for the TGF-β1-induced upregulation of VEGF, a potent and specific TβRI inhibitor, SB431542, was used to block the activation of TβRI. As shown in Fig. 2a, treatment with 10 μM SB431542 abolished the TGF-β1-induced increases in VEGF mRNA levels in SVOG cells. In addition, the TGF-β1-induced secretion of VEGF protein was abolished by the inhibition of TβRI (Fig. 2b). Similar results were also obtained in primary hGL cells (Fig. 2c, d). To exclude the possibility that non-specific effects of the pharmacological inhibitor were the cause of these results, endogenous TβRI and TβRII were knocked down by transfecting cells with specific siRNA targeting TβRI or TβRII. As shown in Fig. 3a, b, TβRI and TβRII siRNA significantly decreased TβRI and TβRII mRNA levels in SVOG cells, respectively. In addition, the TGF-β1-induced upregulation of VEGF mRNA expression was abolished by the knockdown of TβRI and TβRII. Moreover, knockdown of TβRI or TβRII abolished TGF-β1-induced VEGF secretion (Fig. 3c, d).

### SMAD signaling pathways are involved in TGF-β1-induced VEGF expression and secretion

**Fig. 4:**
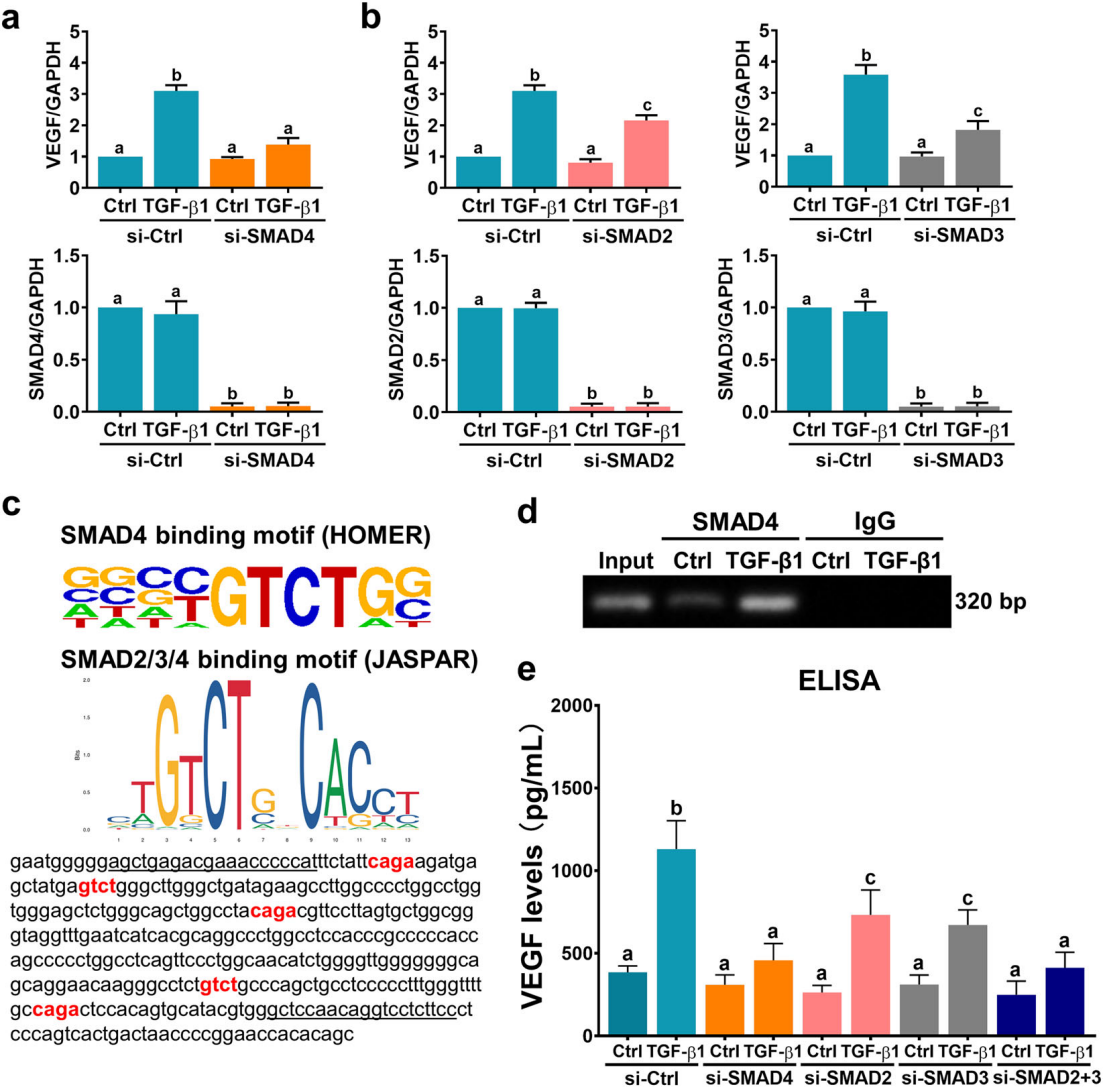
SMAD signaling pathways are involved in TGF-β1-induced VEGF expression and secretion. **a** SVOG cells were transfected with 50 nM control siRNA (si-Ctrl) or SMAD4 siRNA (si-SMAD4), and after 48 h they were treated with 5 ng/mL TGF-β1 for 6 h. VEGF and SMAD4 mRNA levels were examined by RT-qPCR. **b** SVOG cells were transfected with 50 nM control siRNA (si-Ctrl), SMAD2 siRNA (si-SMAD2) or SMAD3 siRNA (si-SMAD3), and after 48 h they were treated with 5 ng/mL TGF-β1 for 6 h. VEGF, SMAD2, and SMAD3 mRNA levels were examined by RT-qPCR. **c** Photo of SMAD-binding motifs in the HOMER database and JASPAR database (upper panel). The SMAD-binding site in the human VEGF promoter is highlighted in red. The primers for the ChIP assay are underlined (lower panel). **d** SVOG cells were treated with 5 ng/mL TGF-β1 for 1 h before being subjected to ChIP analysis. Anti-SMAD4 or IgG antibodies were used to immunoprecipitate DNA-containing complexes. Subsequent PCR was performed with primers that were complementary to the VEGF promoter region and contained the SMAD-binding site. The PCR products were resolved by electrophoresis on a 1% agarose gel and were visualized by ethidium bromide staining. **e** SVOG cells were transfected with 50 nM control siRNA (si-Ctrl), SMAD4 siRNA (si-SMAD4), SMAD2 siRNA (si-SMAD2), SMAD3 siRNA (si-SMAD3) or SMAD2 + SMAD3 siRNAs (si-SMAD2 + 3), and after 48 h they were treated with 5 ng/mL TGF-β1 for 24 h. The protein levels of VEGF in culture media were examined by ELISA. The results are expressed as the mean ± SEM of at least three independent experiments. The values without a common letter are significantly different (*p* < 0.05).

TGF-β1-activated SMAD2 and SMAD3 bind to the common mediator SMAD4. The SMAD complexes then translocate to the nucleus, where they mediate TGF-β1-regulated gene expression^32^. We have shown that treatment of hGL cells with TGF-β1 activates both the SMAD2 and SMAD3 signaling pathways in hGL cells^23,24^. To examine the involvement of SMAD signaling pathways in TGF-β1-induced VEGF expression, an siRNA-mediated approach was used to knock down SMAD4. As shown in Fig. 4a, transfection of SVOG cells with SMAD4 siRNA significantly knocked down the endogenous expression of SMAD4. In addition, the TGF-β1-induced upregulation of VEGF mRNA expression was abolished by SMAD4 knockdown. SMAD2 and SMAD3 are functionally interchangeable and equally important for mediating the biological effects of TGF-β1. However, few studies have suggested that SMAD2 and SMAD3 have distinct and nonoverlapping roles in TGF-β1 signaling^33^. Therefore, we next explored whether SMAD2 and SMAD3 play similar roles in mediating TGF-β1-induced VEGF expression in and secretion from hGL cells. SMAD2 and SMAD3 siRNA were used to knock down endogenous expression of SMAD2 and SMAD3, respectively. As shown in Fig. 4b, knockdown of SMAD2 partially attenuated TGF-β1-induced VEGF expression. Similar results were observed when SMAD3 was knocked down. SMAD proteins bind to the consensus sequences CAGA or GTCT, which give rise to the biological functions of TGF-β1^34,35^. Published SMAD4 and SMAD2/3/4 binding motifs shown that were identified in the HOMER and JASPAR databases were located in the VEGF promoter (Fig. 4c). Then, a ChIP assay was employed to determine whether SMAD4 could directly bind to the VEGF promoter. As shown in Fig. 4d, SMAD4 specifically bound to the VEGF promoter after treatment with TGF-β1 for 1 h. Moreover, ELISA analyses showed that knockdown of SMAD2 or SMAD3 partially attenuated TGF-β1-induced VEGF secretion. In addition, TGF-β1-induced VEGF secretion was abolished by simultaneously knocking down SMAD2 and SMAD3, and the effect was similar after knocking down SMAD4 (Fig. 4e). Taken together, these results indicate that SMAD signaling pathways are involved in TGF-β1-induced VEGF expression and secretion in hGL cells.

### ERK1/2 and p38 MAPK signaling pathways are involved in TGF-β1-induced VEGF expression and secretion

**Fig. 5:**
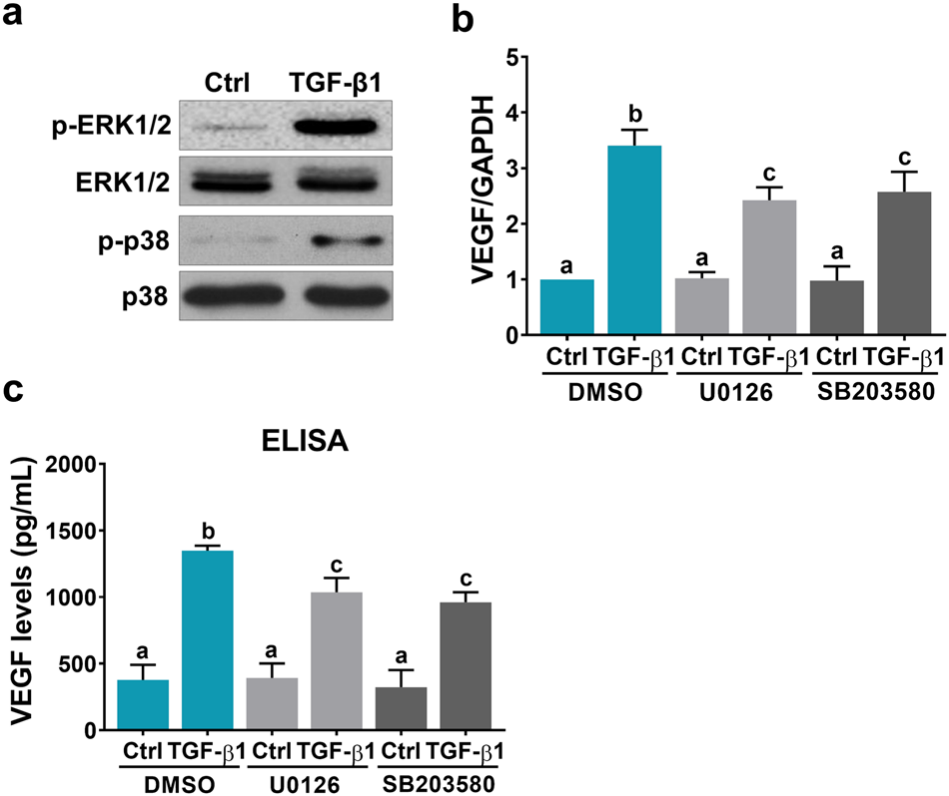
ERK1/2 and p38 MAPK signaling pathways are involved in TGF-β1-induced VEGF expression and secretion. **a** SVOG cells were treated with 5 ng/mL TGF-β1 for 10 min. Phosphorylated and total ERK1/2 and p38 expression levels were determined by western blot. **b** SVOG cells were pretreated with a vehicle control (DMSO), 10 µM U0126 or 10 µM SB203580 for 1 h, and then they were treated with 5 ng/mL TGF-β1 for 6 h. VEGF mRNA levels were examined by RT-qPCR. **c** SVOG cells were pretreated with a vehicle control (DMSO), 10 µM U0126 or 10 µM SB203580 for 1 h, and then they were treated with 5 ng/mL TGF-β1 for 24 h. VEGF protein levels in culture media were examined by ELISA. The results are expressed as the mean ± SEM of at least three independent experiments. The values without a common letter are significantly different (*p* < 0.05).

In addition to the canonical SMAD-dependent pathways, TGF-β1 is able to activate other SMAD-independent noncanonical signaling pathways^36^. It has been shown that TGF-β1 can stimulate VEGF expression in SMAD4-null colon cancer cells by activating the ERK1/2 and p38 MAPK signaling pathways^37^. Consistent with our previous study^23^, we found that TGF-β1 treatment activated the ERK1/2 signaling pathway. In addition, the p38 MAPK signaling pathway was activated upon TGF-β1 treatment in SVOG cells (Fig. 5a). To examine whether these two signaling pathways were involved in TGF-β1-induced VEGF expression, the MEK inhibitor U0126 and the p38 MAPK inhibitor SB203580 were used to block the activation of these pathways. As shown in Fig. 5b, treatment with 10 μM U0126 or SB203580 attenuated the TGF-β1-induced upregulation of VEGF mRNA expression in SVOG cells. In addition, the TGF-β1-induced secretion of VEGF protein was attenuated by the inhibition of the ERK1/2 or p38 MAPK signaling pathway (Fig. 5c). Taken together, these results indicate that the ERK1/2 and p38 MAPK signaling pathways are involved in TGF-β1-induced VEGF expression in and secretion from hGL cells.

### Blocking TGF-β1 signaling attenuates the pathogenesis of OHSS in mice

**Fig. 6:**
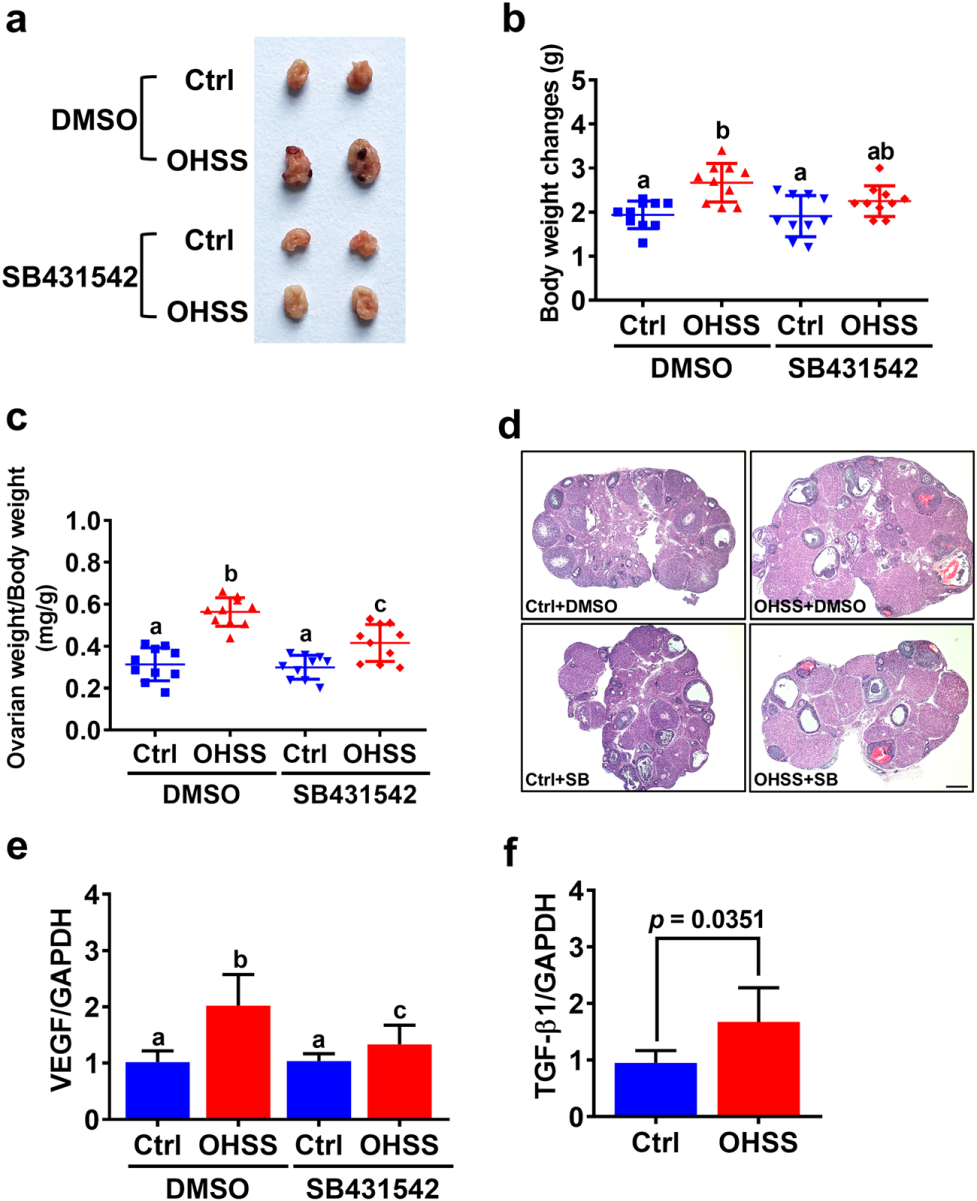
Inhibition of TGF-β1 signaling inhibits the pathogenesis of OHSS. **a** Representative ovaries from each group were photographed. **b** Changes in body weight throughout the treatments were determined after mice were euthanized. **c** Ovarian weight as it related to body weight was determined after mice were euthanized. **d** Images of representative H&E stained ovarian sections. Original magnification. 40×, Scale bars represent 200 μm. **e**, **f** VEGF (**e**) and TGF-β1 (**f**) mRNA levels in mouse ovaries were examined by RT-qPCR. The results are expressed as the mean ± SD. The values without a common letter are significantly different (*p* < 0.05).

To further explore the role of TGF-β1 in regulating OHSS pathogenesis, we used SB431542 to block TGF-β1 function of in a mouse OHSS model. Consistent with previous studies, induction of OHSS significantly increased the body weight, and the size and weight of the ovary^31,38^. Interestingly, administration of SB431542 alleviated the severity of the symptoms in the OHSS group, including increased body weight, and ovary size and weight (Fig. 6a–c). Similar to previous studies^38,39^, histological analysis showed that an increase in the number of corpus lutea was observed in the OHSS group. Blocking TGF-β1 signaling by treatment with SB431542 reduced the number of corpus lutea (Fig. 6d). In addition, RT-qPCR results showed that VEGF mRNA was upregulated in the ovaries of the OHSS mice, but the induction of VEGF mRNA levels was attenuated by the administration of SB431542 (Fig. 6e). Moreover, the mRNA levels of TGF-β1 were also significantly increased in the ovaries of the OHSS mice (Fig. 6f).

### TGF-β1 levels are upregulated in the follicular fluid of OHSS patients and are positively correlated with follicular fluid VEGF levels

**Fig. 7:**
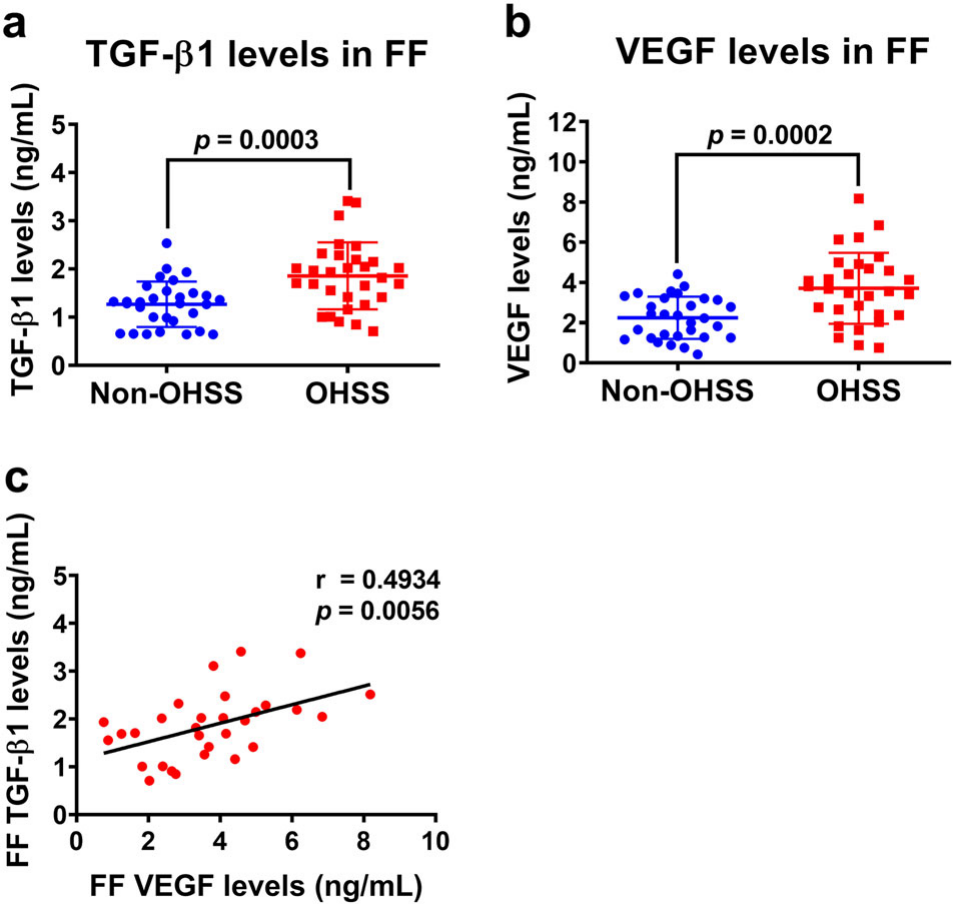
TGF-β1 and VEGF protein levels are increased in the follicular fluid of patients with OHSS. **a**, **b** TGF-β1 (**a**) and VEGF (**b**) protein levels in the follicular fluid of patients without OHSS (*n* = 30) and with OHSS (*n* = 30) were examined by ELISA. The results are expressed as the mean ± SD. The values without a common letter are significantly different (*p* < 0.05). **c** Pearson’s correlation analysis was performed to examine the correlation coefficient between two values. TGF-β1 protein levels were positively correlated with VEGF protein levels in the follicular fluid of patients with OHSS (*n* = 30).

Follicular fluid provides a critically important microenvironment for the development of the ovarian follicle and the oocyte. TGF-β1 and VEGF can be secreted by granulosa cells into the follicular fluid, where they exert their biological effects in an autocrine or paracrine fashion. Therefore, we next examined the TGF-β1 and VEGF levels in the follicular fluid of OHSS patients. Follicular fluid samples from women without OHSS (non-OHSS, *n* = 30) and those with severe OHSS (OHSS, *n* = 30) were collected during oocyte retrieval for IVF treatment. As shown in Fig. 7a, similar to the results obtained from the mouse OHSS model, the protein levels of TGF-β1 were significantly higher in the follicular fluid of OHSS patients than they were in non-OHSS patients. As expected, VEGF protein levels were considerably higher in the follicular fluid of OHSS patients than they were in non-OHSS patients (Fig. 7b). Importantly, Pearson’s correlation analysis showed that the protein levels of TGF-β1 and VEGF in the follicular fluid of OHSS patients were positively correlated (Fig. 7c).

## Discussion

TGF-β1 is a secreted, extracellular growth factor, and accumulating evidence indicates that this protein plays important roles in the regulation of ovarian function^40^. However, much of this evidence is derived from animal models; therefore, a detailed investigation of the roles of TGF-β1 in human ovarian function is required. To the best of our knowledge, there has only been one study that addressed the effect of TGF-β1 on VEGF expression in ovarian granulosa cells. In that study, the VEGF expression in and secretion from rat granulosa cells were increased after treatment with TGF-β1, which, in turn, contributed to upregulated angiogenic activity^26^. In the present study, we were the first to demonstrate the stimulatory effect of TGF-β1 on VEGF expression and secretion in hGL cells. It has been reported that TGF-β1 can stimulate VEGF expression in and secretion from different types of normal cells^41–43^. Interestingly, treatment of human keratinocytes with TGF-β1 antisense oligonucleotides induced VEGF expression^44^. These studies indicate that the effect of TGF-β1 on VEGF expression could be cell-type-dependent.

Using SMAD2- and SMAD3-deficient mouse embryonic fibroblasts as experimental models, TGF-β1-induced VEGF expression was found to be mediated by both SMAD2 and SMAD3^43^. Intriguingly, using the same cell models, the stimulatory effect of TGF-β1 on VEGF expression is blocked in SMAD3-deficient cells but not in SMAD2-deficient cells^45^. In the present study, instead of using SMAD-deficient cell models, we used an siRNA-mediated knockdown approach to examine the involvement of SMAD2 and SMAD3 in TGF-β1-induced VEGF expression in and secretion from hGL cells. Our results clearly showed that knockdown of SMAD2 or SMAD3 partially attenuated the induction of VEGF expression and secretion by TGF-β1, indicating that both SMAD2 and SMAD3 are required for TGF-β1-induced VEGF expression and secretion. Upon TGF-β1 binding, the activated TGF-β receptors induce phosphorylation of the receptor-regulated SMAD proteins SMAD2 and SMAD3. Phosphorylated SMAD2 or SMAD3 consequently binds to the common mediator SMAD4, and the SMAD complex subsequently translocates to the nucleus to mediate TGF-β1-regulated gene expression^32^. Our ChIP results with a SMAD4 antibody indicated that SMAD complexes can bind directly to the VEGF promoter. It has been shown that the DNA binding affinity of activated SMAD complexes is low and is not strong enough to maintain the association with endogenous promoters of target genes. SMAD complexes usually interact with other DNA binding cofactors to achieve high affinity and selectivity for target promoters of genes^46^. Among these DNA binding cofactors, some have been reported to be involved in TGF-β1-induced VEGF expression^47^. Whether the transcriptional regulatory machinery is the same in human granulosa cells remains unknown and warrants future research. In addition to SMAD pathways, TGF-β1 has been shown to upregulate VEGF expression in colon cancer cells by activating ERK1/2 and p38 MAPK signaling pathways^37^. Similar to this previous study, our results showed that the ERK1/2 and p38 MAPK signaling pathways were activated by TGF-β1 and that both were involved in TGF-β1-induced VEGF expression and secretion. Taken together, in hGL cells, the SMAD2/3, ERK1/2 and p38 MAPK signaling pathways are required for TGF-β1-induced VEGF expression and secretion.

Although the incidence of severe OHSS is low, this disease remains a serious complication of in vitro fertilization treatment. Due to its elusive pathophysiology, clinically, OHSS has mainly been managed expectantly and empirically. Thus far, several approaches have been used to prevent the occurrence of OHSS^1^. Given the pivotal role of VEGF in the pathogenesis of OHSS, targeting VEGF has been employed to prevent the occurrence of OHSS^20^. In the present study, our in vitro data as well as our animal and clinical results demonstrated that TGF-β1 was upregulated in the mouse ovary and follicular fluid of patients with OHSS, and this upregulation subsequently contributes to increased VEGF expression. Importantly, blocking TGF-β1 signaling attenuated the pathogenesis of OHSS by decreasing VEGF expression in the mouse OHSS model. These results suggest that targeting TGF-β1 signaling could be an alternative approach for preventing the development of OHSS. To the best of our knowledge, to date, the regulation of TGF-β1 expression in granulosa cells is largely unknown. It has been shown that endoplasmic reticulum (ER) stress induces TGF-β1 expression in hGL cells^48^. Interestingly, ER stress stimulates VEGF expression in hGL cells, and inhibition of ER stress decreases VEGF expression in a rat OHSS model^49^. These studies suggest that ER stress-induced TGF-β1 may contribute to VEGF expression in OHSS. Further investigations into other factors that contribute to the upregulation of TGF-β1 expression in the human granulosa cells of OHSS are urgently needed. In addition, whether the expression levels of TβRI and TβRII are varied in granulosa cells between patients with and without OHSS requires further investigation.

In summary, the present study clearly demonstrates the stimulatory effect of TGF-β1 on VEGF expression in and secretion from hGL cells. Our results reveal that the SMAD2/3, ERK1/2, and p38 MAPK signaling pathways are involved in TGF-β1-stimulated VEGF expression and secretion. Importantly, for the first time, we show that the expression of TGF-β1 was increased in the ovaries of OHSS mice. In addition, blocking TGF-β1 signaling inhibited the pathogenesis of OHSS in mouse OHSS model by attenuating the expression of VEGF. Clinically, the protein levels of TGF-β1 and VEGF were both increased in the follicular fluid of OHSS patients. The expression levels of TGF-β1 and VEGF in the follicular fluid were positively correlated. These results provide a better understanding of the mechanisms mediating the expression of VEGF in hGL cells, which could lead to the development of alternative therapeutic approaches for OHSS.

## Supplementary information

Supplemental Table 1
