# Supplementary figures and images for: TGF-β1 induces VEGF expression in human granulosa-lutein cells: a potential mechanism for the pathogenesis of ovarian hyperstimulation syndrome
Source: Exp Mol Med. 2020 Mar 10;52(3):450–60. doi: 10.1038/s12276-020-0396-y (PMC7156760; doi:10.1038/s12276-020-0396-y)

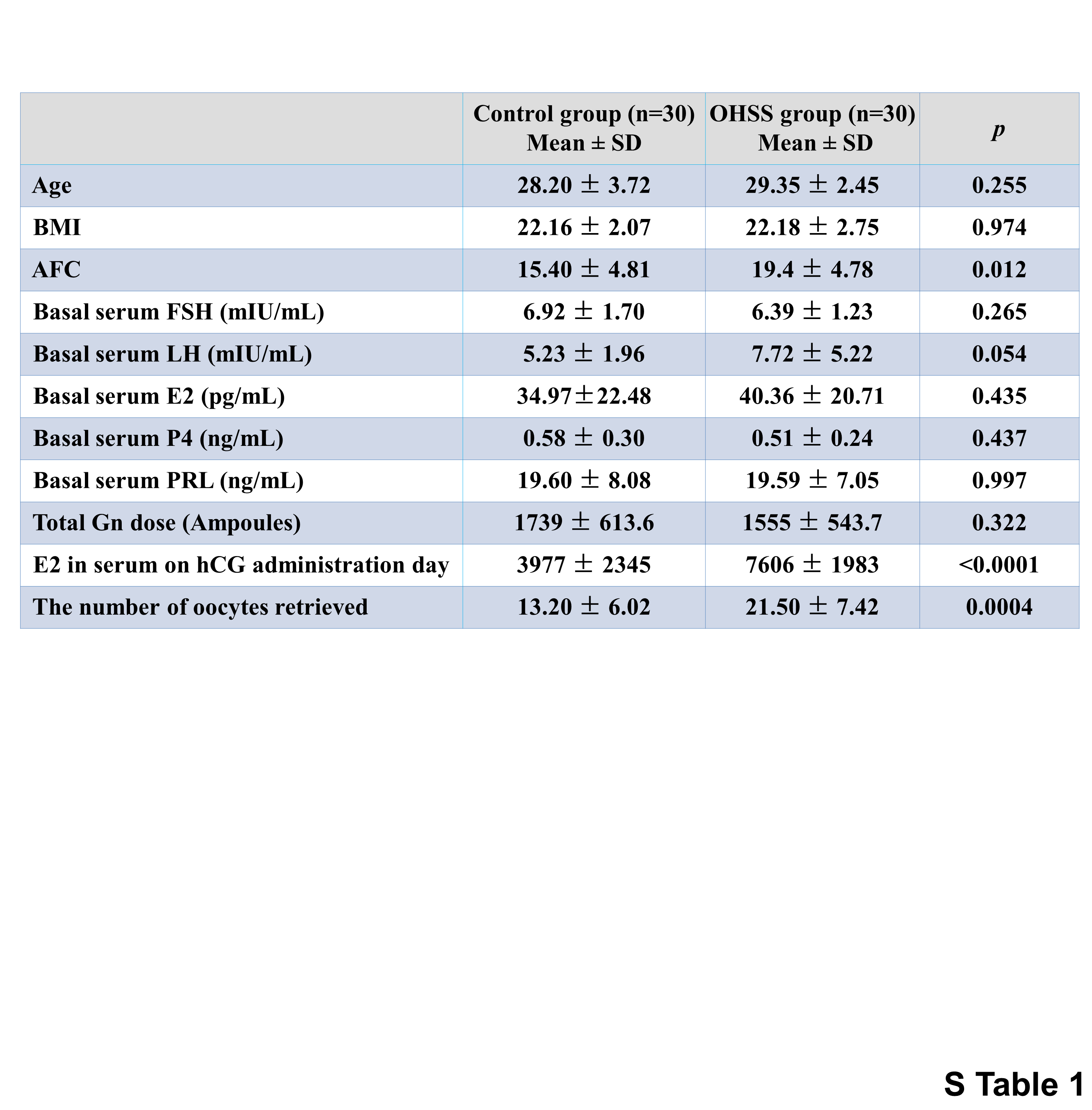

Supplement: Supplementary file 1 — Supplemental Table 1 [file 12276_2020_396_MOESM1_ESM.tif]
